# Data-Driven and Multiscale Modeling of DNA-Templated Dye Aggregates

**DOI:** 10.3390/molecules27113456

**Published:** 2022-05-27

**Authors:** Austin Biaggne, Lawrence Spear, German Barcenas, Maia Ketteridge, Young C. Kim, Joseph S. Melinger, William B. Knowlton, Bernard Yurke, Lan Li

**Affiliations:** 1 Micron School of Materials Science and Engineering, Boise State University, Boise, ID 83725, USA; austinbiaggne@u.boisestate.edu (A.B.); lawrencespear@boisestate.edu (L.S.); germanbarcenas@u.boisestate.edu (G.B.); maiaketteridge@u.boisestate.edu (M.K.); bknowlton@boisestate.edu (W.B.K.); bernardyurke@boisestate.edu (B.Y.); 2 Materials Science and Technology Division, U.S. Naval Research Laboratory, Washington, DC 20375, USA; youngchan.kim@nrl.navy.mil; 3 Electronics Science and Technology Division, U.S. Naval Research Laboratory, Washington, DC 20375, USA; joseph.melinger@nrl.navy.mil; 4 Department of Electrical and Computer Engineering, Boise State University, Boise, ID 83725, USA; 5 Center for Advanced Energy Studies, Idaho Falls, ID 83401, USA

**Keywords:** dye aggregates, DNA scaffolds, exciton, extinction coefficient, transition dipole moment, machine learning, density functional theory, time-dependent density functional theory, molecular dynamics

## Abstract

Dye aggregates are of interest for excitonic applications, including biomedical imaging, organic photovoltaics, and quantum information systems. Dyes with large transition dipole moments (μ) are necessary to optimize coupling within dye aggregates. Extinction coefficients (ε) can be used to determine the μ of dyes, and so dyes with a large ε (>150,000 M^−1^cm^−1^) should be engineered or identified. However, dye properties leading to a large ε are not fully understood, and low-throughput methods of dye screening, such as experimental measurements or density functional theory (DFT) calculations, can be time-consuming. In order to screen large datasets of molecules for desirable properties (i.e., large ε and μ), a computational workflow was established using machine learning (ML), DFT, time-dependent (TD-) DFT, and molecular dynamics (MD). ML models were developed through training and validation on a dataset of 8802 dyes using structural features. A Classifier was developed with an accuracy of 97% and a Regressor was constructed with an $R^{2}$ of above 0.9, comparing between experiment and ML prediction. Using the Regressor, the ε values of over 18,000 dyes were predicted. The top 100 dyes were further screened using DFT and TD-DFT to identify 15 dyes with a μ relative to a reference dye, pentamethine indocyanine dye Cy5. Two benchmark MD simulations were performed on Cy5 and Cy5.5 dimers, and it was found that MD could accurately capture experimental results. The results of this study exhibit that our computational workflow for identifying dyes with a large μ for excitonic applications is effective and can be used as a tool to develop new dyes for excitonic applications.

## 1. Introduction

Organic molecules, which absorb and emit light, also known as dyes, are useful for many applications, such as biomedical imaging [1,2], organic photovoltaics [3,4], non-linear optics [5], and quantum information systems [6,7,8,9]. Key parameters that determine the performance of the dyes in those applications include the extinction coefficient (ε) and transition dipole moment (μ), as well as aggregation ability. Thus, optimizing the key electronic (e.g., μ) and molecular (e.g., aggregate) features is crucial for the desired applications of dye molecules. The interaction of dyes with light can be quantified via their extinction coefficient, ε. The value of ε, resulting from the absorption of light by the dye, can be measured using optical spectroscopy. From the measured value of ε, the transition dipole moment μ can be extracted [10,11,12]. This relationship not only allows for the measurement of the μ of dyes, but also helps select the dye candidates with optimal electronic properties for excitonic applications (i.e., large μ) from numerous dyes. The value of ε strongly depends on the molecular structure of the dye. Some efforts have been made to augment the ε of dyes, such as adding donor or acceptor groups to the π-conjugation network [13], extending the π-conjugation network [14], and making the dye structures more planar [15,16,17]. However, the relationship between dye structure and ε remains unclear. In addition, it is time-consuming to conduct either experimental measurements or computational modeling to screen many dye candidates for desirable properties (e.g., high ε and μ).

Another key feature of dyes for excitonic applications is dye aggregation. Dye aggregation has been observed in natural systems [18,19] as well as artificial systems [20,21]. Dye aggregates feature exciton delocalization, which facilitates energy transfer through the aggregate [22]. The dynamics of excitons residing on a dye aggregate can be described using the Frenkel Hamiltonian [23], where the exchange of excitons is largely dependent on the transition dipole coupling of the dyes [22,23,24]. The dipole coupling strength, exciton delocalization, and corresponding dynamics depend on the electronic properties of individual dyes, or monomers, as well as the orientations of the dyes in the aggregate [22,23,24,25,26]. One method of facilitating dye aggregation in a controlled and predictable manner is using DNA scaffolds. Dyes attached to DNA scaffolds, such as duplexes, Holliday junctions, and origami, have been shown to aggregate into dimers, trimers, and tetramers [27,28,29,30,31,32,33,34,35,36,37,38,39]. Characterization of the optical properties of the aggregates reveals that the dyes can adopt various ideal orientations [22,24,26,40]. One orientation, called an H-aggregate, occurs when the dyes are stacked, and results in a blue-shifted absorption spectrum. When the dyes are oriented head-to-tail, called a J-aggregate, the absorption spectrum is red-shifted. Another aggregate, termed oblique, occurs when the dyes are at 90° to one another, and results in Davydov splitting of the absorption spectrum. To maximize or fine-tune the coupling between dye molecules, the aggregation should be predictable and controlled.

Computational studies of dyes aim to identify optimal candidates for excitonic applications. For example, density functional theory (DFT) and time-dependent (TD-) DFT can be used to screen the effects of functional groups on dye electronic properties [41,42,43,44]. Our prior studies indicated that functional group substitution can affect the solvation free energy ${\Delta G}_{\mathrm{solv}}$, transition dipole moment μ, and absorption wavelength $\lambda_{\max}$ of a dye [45,46]. This effect is correlated with the empirically derived Hammett constant ($\sigma_{p}$), which demonstrates the electron-donating or electron-withdrawing strength of a substituent. The DFT and TD-DFT methods are applicable to dye monomers, but not to dye aggregates attached to DNA scaffolds due to structural size and complexity. An alternative method to further screen dyes with favorable electronic properties attached to DNA scaffolds is molecular dynamics (MD), which has been used to study dye–DNA interactions [47,48,49,50]. In a recent study, Mathur et al. used MD to study the orientations of cyanine dyes attached to DNA bundles and found that they were able to accurately capture dye dynamics and orientations using MD [47]. Nicoli et al. utilized MD to study the aggregation of Cy3 dimers attached to DNA duplexes, and found that MD could accurately capture the stacking of dyes leading to H-aggregation [50]. However, both DFT and MD are time-consuming for high-throughput screening of dye candidates with desired properties. Recently, machine learning (ML) has been shown to be a viable method of screening thousands of molecules to identify structure–property relationships based on both computational (e.g., DFT) [51] and experimental data [52,53]. The problem of searching through chemical-based datasets that contain labeled data for optimal molecules is a common task for pharmaceuticals and dye-sensitized solar cells, but there has not yet been work specifically targeting optimization of dye candidates for dye aggregate–DNA constructs. In particular, our group is interested in near-IR dye molecules exhibiting a large ε (>150,000 M^−1^cm^−1^) and hydrophobic properties. The same photophysical data used to create the chemical-based datasets for dyes have been of interest for organic photovoltaics, and there are several public datasets available [52,53,54,55]. ML techniques applied to chemical space exploration is a rich field with a variety of methods from which to choose. The methods can be hierarchical with the size of the dataset, spanning from well-established supervised learning to more complex artificial neural networks [56].

In this work, a systematic approach, combining ML, DFT, TD-DFT, and MD methods, was used to screen dye monomers from an expansive dataset and provide insight into dye aggregate–DNA duplex interactions. We first used ML to identify ideal dye candidates with high extinction coefficients (ε) from a dataset of around 18,000 molecules. Then, for the 100 ML-selected dye candidates with desirable structural features and high values of ε, DFT and TD-DFT calculations were performed to predict their ground and excited state properties. Finally, benchmark MD simulations were conducted to reveal the interactions between the selected dye dimers and the DNA duplexes.

## 2. Methods

### 2.1. Machine Learning

**Figure 1:**
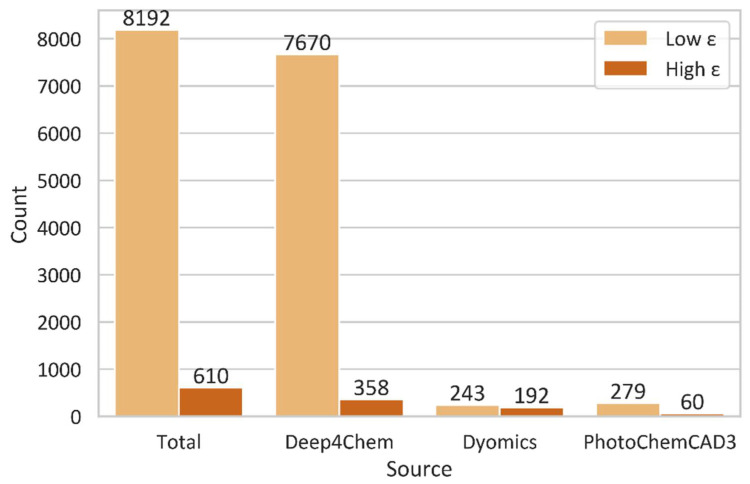
Dataset breakdown for molecules with high and low extinction coefficients ε. The threshold is 150,000 M^−1^cm^−1^.

Classifier and Regressor models were trained to identify ideal dye candidates with high extinction coefficients (ε) based on dye structure features. The Classifier model could quickly classify the dyes with either high or low ε, where we set a threshold of 150,000 M^−1^cm^−1^ for strong exciton coupling in dye aggregates. As it learned from the Classifier model, the Regressor model could further estimate the values of ε for the dyes. Three data sources, including Deep4Chem [57], PhotoChem CAD 3 [54], and Dyomics GmbH [58] (8802 molecule datapoints in total), were used to train and validate the models. We utilized SMILES format for the molecule. We also utilized RDKit [59] to calculate 284 different features, such as the maximum carbon chain length and aromatic, amide, and ester group counts. The development dataset, containing 90% of data points, was used to train and test various model hyperparameters. Based on the accuracy for the Classifier and R^2^ for the Regressor, a model was selected for further analysis. We then used the validation dataset, containing 10% of data points, to validate the selected model’s effectiveness. The molecules with ε of above 800,000 M^−1^cm^−1^ were excluded because the ε values deviated too greatly from the threshold of 150,000 M^−1^cm^−1^. Figure 1 shows the dataset breakdown with high and low ε values. The three data sources all have a data imbalance, where the number of molecules with low ε values is larger than that of molecules with high ε values. The effect of imbalanced data is discussed in Section 3.1.

### 2.2. Density Functional Theory

Density functional theory (DFT) and time-dependent (TD-) DFT calculations were performed to optimize dye structures in the ground state and calculate solvation energies and transition dipole moments. Similarly to our previous work [46], the dyes were optimized with the M06-2X [60] functional and 6-31+G(d,p) basis set, to a residual force of 4.5 × 10^−4^ Hartree/Bohr. The M06-2X functional with 6-31+G(d,p) basis set was validated in our prior studies of pristine and substituted cyanine and squaraine dyes [45,46] and has been used successfully for the calculations of the excited state properties of similar systems [61,62,63]. Frequency calculations were conducted to confirm the ground state structures were true minima. To determine μ, single point, vertical excited state calculations using the M06-2X functional were performed on the ground state structures to obtain transitions to the first 30 excited singlet states and identify the state with the largest oscillator strength. Calculations of the ground and excited state properties were conducted with implicit water solvation using the integral equation formalism polarizable continuum model (IEFPCM) [64,65], which was successfully used for the excited state property calculations of similar systems [66,67,68]. Excited state calculations were conducted assuming nonequilibrium solvent conditions.

To approximate the relative hydrophobicity of the dyes, the partitioning coefficient between n-octanol and water, $\log\left(P_{o/w}\right)$, was calculated according to [69,70]

(1) $$\log\left(P_{o/w}\right)=-\frac{{\Delta G}_{o}-{\;\Delta G}_{w}}{2.3\mathrm{RT}}$$

where ${\Delta G}_{o}$ and ${\Delta G}_{w}$ are the Gibbs free energy of solvation for a dye in n-octanol and water, respectively; $R\;=8.31\frac{J}{\mathrm{mol}\cdot K}$; and T =273.15 K. A more positive value of $\log\left(P_{o/w}\right)$ means a molecule is more hydrophobic, and a more negative value means a molecule is more hydrophilic. In general, the Gibbs free energy of solvation for a molecule (${\Delta G}_{\mathrm{solv}}$) is a measure of the amount of energy required to dissolve the dye in solvent, and was calculated according to [45,46,68,71]

(2) $${\Delta G}_{\mathrm{solv}}={\;E}_{\mathrm{solvated}}-{\;E}_{\mathrm{vacuum}}$$

where $E_{\mathrm{solvated}}$ is the total energy of the dye in implicit solvent and $E_{\mathrm{vacuum}}$ is the total energy of the dye in vacuum. Calculations for the solvation energy were conducted using the universal solvation model based on density (SMD) variation of IEFPCM [72], which was useful for predicting the solvation energies of organic molecules [73] and calculating the relative hydrophobicity of modified squaraine dyes [70]. All DFT and TD-DFT calculations were conducted using the Gaussian16 software package [74].

### 2.3. Molecular Dynamics

Molecular dynamics (MD) simulations were performed with the GROMACS 2020.3 software package [75]. Dye–DNA structures were built using the UCSF ChimeraX software [76] with the dyes initialized on the outside of the DNA backbone. The OL15 force-field [77] with non-bonded modifications [78] was used for DNA parameters and the generalized amber forcefield (GAFF) [79] was used for dye parameters. Atomic charges for the dyes were calculated using the HF/6-31G* theory level [80]. The 26-basepair dsDNA duplex sequence and dye locations from Huff et al. [29] was used. The dye–DNA structures were solvated in TIP3P water [81] in a truncated octahedron box with 1.2 nm between the dye–DNA structure and the box edge. Mg^2+^ ions were used to neutralize the system. Cannon et al. and Huff et al. showed, experimentally, that by adding excess MgCl_2_ to solutions containing DNA duplexes with two pentamethine indocyanine Cy5 dyes, DNA Holliday junctions could be formed [28,29]. Because of this, no excess MgCl_2_ was used in the MD simulations apart from replacing a necessary number of water molecules with Mg^2+^ to achieve neutral charge. Neighbor-searching was used with a cutoff of 1.2 nm. Van der Waals interactions were limited to 1.2 nm, and the particle mesh Ewald (PME) was used with a real-space coulomb cutoff of 1.2 nm. Bonds to hydrogen atoms were constrained using the LINCS algorithm [82]. A timestep of 2 fs was used.

The initial systems were energy-minimized with the steepest descent method for 1000 steps. Then, to achieve a well-relaxed starting structure, two subsequent 10 ns equilibration steps were performed with harmonic constraints, the first with 1000 $\frac{\mathrm{kJ}}{\mathrm{mol}\cdot {\mathrm{nm}}^{2}}$ spring constants applied to non-hydrogen atoms, and the second with 100 $\frac{\mathrm{kJ}}{\mathrm{mol}\cdot {\mathrm{nm}}^{2}}$ spring constants applied to non-hydrogen atoms, keeping the number of atoms, volume, and temperature constant. A final 10 ns equilibration was performed with no restraints. Following equilibration, 1 μs production simulations were carried out, keeping the number of atoms, pressure, and temperature constant. The velocity-rescale thermostat [83] was used to maintain a constant temperature of 300 K with a coupling time of 0.1 ps, with the DNA–dye and solvent being coupled separately. The Parrinello–Rahman barostat [84] was used to keep the pressure at 1 atm with a coupling time of 1.0 ps. Coordinates were written every 10 ps and the first 100 ns of the production simulations were treated as equilibration periods, and so were not used for analysis.

To determine the transition dipole coupling strength between two dyes, the orientation of the dyes with respect to one another and the transition dipole moments μ are needed. The dye–dye center-to-center distances $(R_{m,n})\;$ and orientation factors (κ) were determined every 10 ps. The values of κ were determined using [48]

(3) $$\kappa \;={\hat{\mu }}_{m}\cdot {\hat{\mu }}_{n}-3\left({\hat{R}}_{m,n}\cdot {\hat{\mu }}_{m}\right)\left({\hat{R}}_{m,n}\cdot {\hat{\mu }}_{n}\right)$$

where ${\hat{\mu }}_{i}$ is the transition dipole moment unit vector of dye m or n (taken along the long axis of the dye), and ${\hat{R}}_{m,n}$ is the unit vector between the centers of dyes m and n. When |κ|=0 or |κ|=1.5, the dyes are in a stacked oblique orientation or tail-to-tail oblique orientation, respectively. When |κ|=1 or |κ|=2, the dyes are in a stacked (H-aggregate) or head-to-tail (J-aggregate) orientation, respectively.

The exciton exchange energy ($J_{m,n}$), which is a measure of the strength of the transition dipole coupling between two dyes, depends on the transition dipole moment μ, which is related to ε [10,11,12] and can be obtained experimentally or using TD-DFT. The $J_{m,n}$ of a dimer was approximated using the extended dipole model according to [85]

(4) $$J_{m,n}={\;J}_{0}\left(\frac{1}{\left|r_{m}-{\;r}_{n}\right|}-\frac{1}{\left|r_{m}-{\;s}_{n}\right|}-\frac{1}{\left|s_{m}-{\;r}_{n}\right|}+\frac{1}{\left|s_{m}-{\;s}_{n}\right|}\right)$$

where the values $r_{i}$ and $s_{i}$ correspond to either end of dye m or n along the dye’s long axis. The pre-factor term, $J_{0}$, is defined as [85]

(5) $$J_{0}=\frac{\mu_{m}\mu_{n}}{4\pi \epsilon_{o}n^{2}l_{m}l_{n}}$$

where $\mu_{i}$ is the transition dipole moment magnitude (calculated using TD-DFT) of dyes m or n, $\epsilon_{0}$ is the vacuum permittivity constant, n is the refractive index of water (1.33), and $l_{m}$ and $l_{n}$ are the lengths of dyes m and n, respectively (such that $l_{i}=\left|r_{i}-{\;s}_{i}\right|$).

## 3. Results

### 3.1. Dye Screening Using Machine Learning and Density Functional Theory

**Figure 2:**
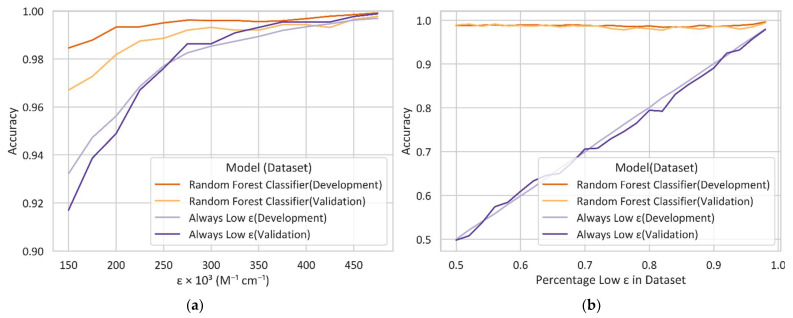
Accuracy comparison between Random Forest Classifier and a model that always classifies a molecule with low ε no matter what structural features the molecule has. They both were developed and validated with (**a**) all and (**b**) partial data.

A Random Forest Classifier was trained on the development dataset utilizing a five k-fold to determine the best ε threshold; the max feature, which refers to the maximum number of features to consider; the max depth, which refers to the maximum depth of the tree; and the class weight for the model. Every model we created has a high accuracy of 97% or above. Figure 2a shows the accuracy of the Random Forest Classifier with various ε thresholds in comparison with a model (labeled Always Low ε) that always classifies a molecule with low ε. The Always Low ε model has a high accuracy of around 92% or above, indicating that the high accuracy of 97% or above for the Random Forest Classifier is reasonable. In addition, the accuracy of the Random Forest Classifier starts to converge at 150,000 M^−1^cm^−1^, which supports our selection of the threshold of 150,000 M^−1^cm^−1^. To address a concern of the effect of imbalanced data on the accuracy of the models, we also trained and validated the models with different datasets that included various percentages of molecules with low ε values. Figure 2b shows the accuracy vs. percentage of low ε in the dataset. The value of 0.5 represents the balanced dataset containing 50% molecules with high ε values and 50% molecules with low ε values. The number of molecules with low ε values gradually increases, so the dataset becomes imbalanced. However, the accuracy of the Random Forest Classifier remains at 97% or above. These results indicate that the effect of the imbalanced data we used to train and validate the models is negligible.

**Figure 3:**
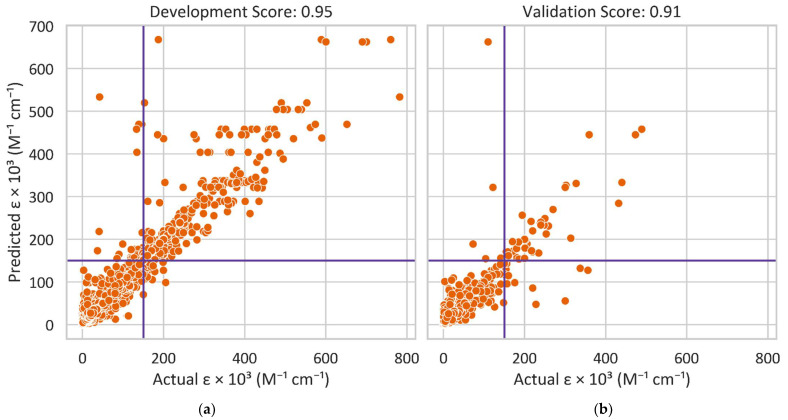
The extinction coefficients ε of the (**a**) development and (**b**) validation datasets predicted by a Random Forest Regressor in comparison with actual values from literature.

Based on the performance of the Random Classifier model, we developed a Random Forest Regressor model to further predict the precise ε value of a molecule. We trained the Regressor to determine its best hyperparameters of class weight, max features, max depth, and criterion as we did with the Random Forest Classifier. Figure 3 compares the predicted and actual ε values for the development and validation datasets, which have 0.95 and 0.91 for R^2^ (i.e., the coefficient of determination), respectively. The datasets and codes developed in this study will be published online.

**Figure 4:**
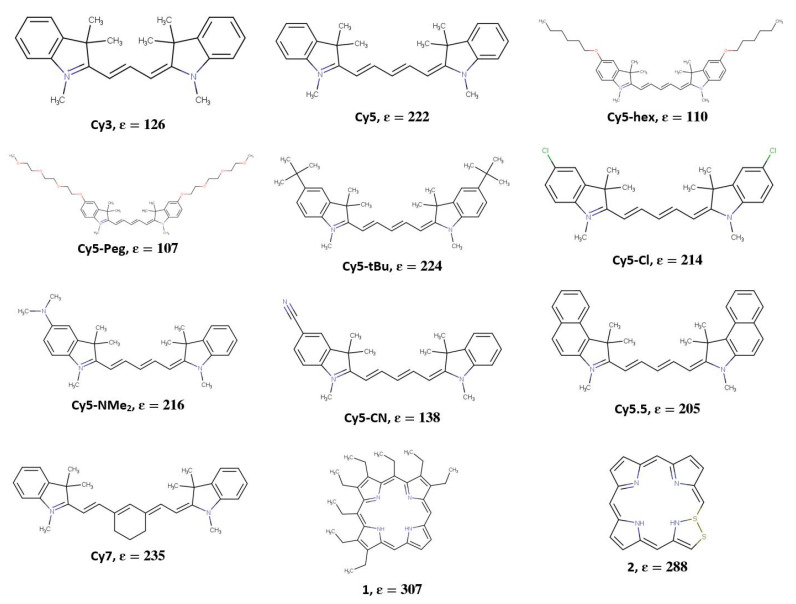

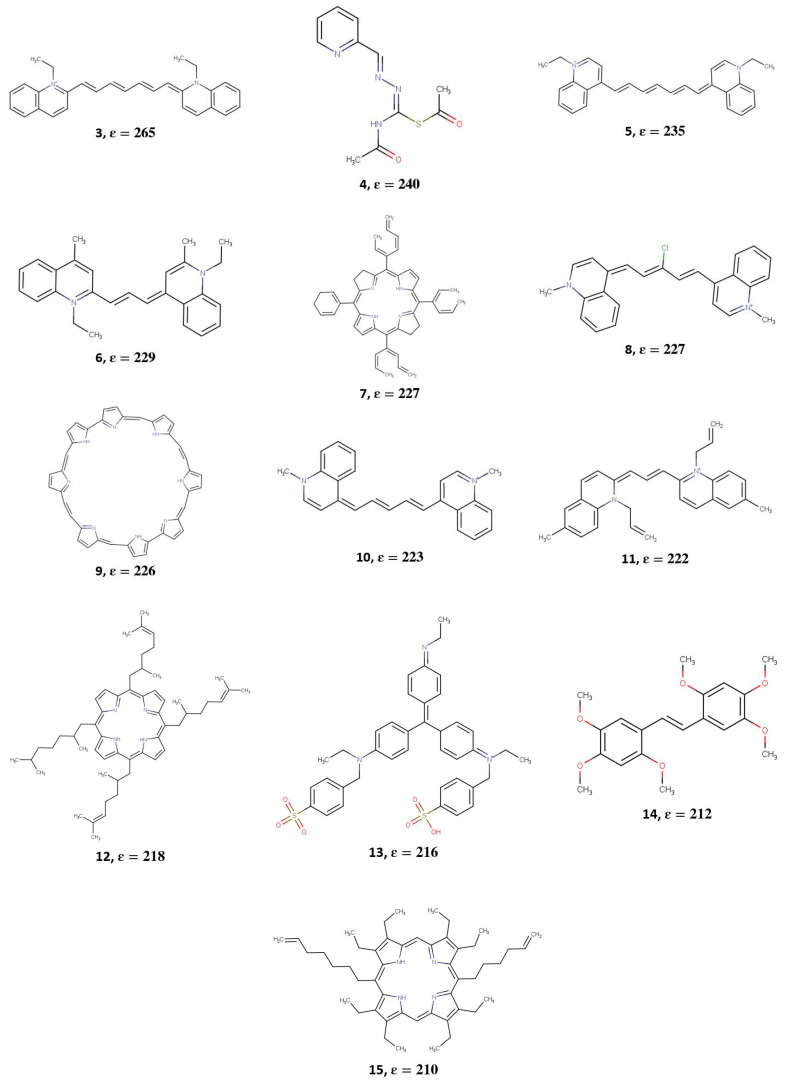
Chemical structures of dyes used in the present study and their machine learning (ML)-predicted extinction coefficients (ε) in units of ×1000 M^−1^cm^−1^. Dyes 1–15 were selected using a combination of ML, density functional theory (DFT), and time-dependent (TD)-DFT.

To identify dyes with high ε (which is indicative of high μ) the optimized Regressor model was further applied to a dataset containing around 18,000 potential dye candidates, including the commercially available cyanine dyes such as Cy3, Cy5, Cy5.5, and Cy7, shown in Figure 4. These dyes are known to exhibit large ε (and thus, large μ and strong dye coupling) [86], and are of interest to our research group and collaborators [27]. Four modified Cy5 dyes with hydrophobic substituents were also considered in this study. These dyes, labeled as Cy5-Cl, Cy5-Peg, Cy5-hex, and Cy5-tBu, are also shown in Figure 4 and are hypothesized to exhibit stronger dye coupling due to being more hydrophobic compared to Cy5, which may result in shorter inter-dye distances [87]. Two other modified dyes, Cy5-CN and Cy5-NMe_2_, were considered since CN and NMe_2_ were shown to have a large effect on excess dipole moments (the difference in the dipole moments of the ground and excited states) of similar dyes [45,46]. The rest of the dataset consisted of dyes obtained from PubChem [88], including dyes with similar structures to cyanine, porphyrin, and methyl violet molecules. Those three classes of dyes were chosen for their prominent π-conjugation [89,90,91], absorption in the visible light range [90,92,93], and excitonic applications [90,92,94,95,96].

**Figure 5:**
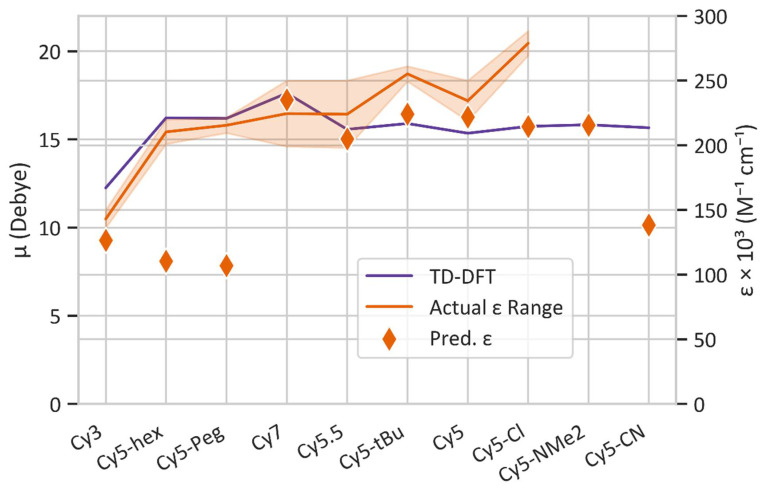
ML-predicted extinction coefficients ε and TD-DFT-calculated transition dipole moments μ of 10 dye candidates of interest in comparison with the experimentally available ε values [29,87,97,98,99,100].

While μ can be extracted from experimental absorption spectra [10,11,12], it is not possible to determine μ based on the peak ε alone. Because of this, TD-DFT was used to calculate μ, to compare with experimentally available ε. Figure 5 shows a comparison of ML-predicted ε and TD-DFT-calculated μ with commercially available Cy3, Cy5, Cy5.5, and Cy7 dyes, as well as modified Cy5 dyes for which experimentally measured values of ε are available. The ε values for the commercially available dyes were obtained from their respective commercial websites, including AAT Bioquest [97], Lumiprobe [98], Glen Research [99], and Interchim [100], and from Huff et al. [29]. Because multiple vendors advertise slightly different ε values, a range is given in Figure 5. Meares et al. synthesized Cy5-hex, Cy5-Peg, Cy5-tBu, and Cy5-Cl for incorporation into DNA and measured the ε of the dyes incorporated into DNA strands at their peak wavelengths [87]. The ranges of ε for Cy5, Cy5-hex, Cy5-Peg, Cy5-tBu, and Cy5-Cl are likely due to small differences in local environments when the dyes are attached to DNA sequences and relative purities [87]. In general, ML-predicted ε values agree with the trend of experimental ε obtained from literature. Notably, ML-predicted ε values for Cy5, Cy5.5, and Cy7 are within the experimental ε range indicated by the shaded region in Figure 5. Similarly, the ML-predicted ε trend agrees with the trend of TD-DFT-calculated μ values, which are assumed to be correlated (i.e., larger ε leads to larger μ). The dyes that do not fall into the range of experimental ε consist of the Cy5 derivatives developed by Meares et al. [87]. The percent errors from the experiments include 45% for Cy5-hex, 49% for Cy5-Peg, 10% for Cy5-tBu, and 20% for Cy5-Cl. Such differences could be caused by solvent, DNA-dye interactions, and dye purities [87]. Furthermore, the specific functional groups for Cy5-hex and Cy5-Peg might not be well represented by the ML training dataset, which could lead to inaccuracies when predicting ε. In general, the Regressor is able to predict the overall trend of ε, which is necessary for the screening of numerous new dyes for ε.

The Regressor model was also applied to the dataset obtained from PubChem [88] to identify additional potential dye candidates. The top 100 dyes that were predicted to have ε above 150,000 M^−1^cm^−1^ were then screened using DFT and TD-DFT to determine their μ values by calculating vertical excited state transitions to the lowest 30 excited states. Of the 100 dye candidates, the 15 dye candidates with desirable properties, such as absorption wavelength in the visible region, large π-conjugated networks, and μ comparable to that of Cy5 (within 50%), are shown in Figure 5 and are labeled 1–15. Their corresponding ML-predicted ε and TD-DFT-calculated μ values are listed in Table 1.

Comparing the TD-DFT calculated values of μ for Cy3, Cy5.5, Cy7, and the Cy5 derivatives, Cy3 has the lowest μ. Disregarding Cy5-hex and Cy5-Peg, the values of μ and the ML-predicted ε generally follow the same trend, with Cy3 having the smallest μ and Cy7 having the largest. Comparing the 15 selected dyes from the Regressor model, all dyes are predicted to have an ε above 210,000 M^−1^cm^−1^. Dye 1 has the largest overall ML-predicted ε of 309,000 M^−1^cm^−1^, with a TD-DFT μ of 9.08 D. Dye 3 has the largest overall TD-DFT μ of 20.25 D and an ML-predicted ε of 265,000 M^−1^cm^−1^.

**Figure 6:**
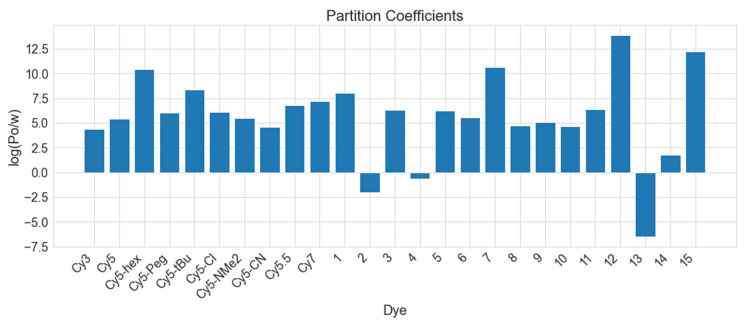
Partition coefficients of dyes in water versus n-octanol (log(P_o/w_)), calculated using Equation (1), where Gibbs free energies of solvation of dyes in implicit water and n-octanol solvents are provided in Table S2. A more positive log(P_o/w_) means a molecule is more hydrophobic, and a more negative log(P_o/w_) means a molecule is more hydrophilic. The labels 1–15 and dye names correspond to the dye structures in Table 1.

Figure 6 shows the log(P_o/w_) values calculated with vacuum and implicit solvent DFT for the ML-selected dyes. A more positive value of log(P_o/w_) indicates a more hydrophobic dye, and a more negative value of log(P_o/w_) indicates a more hydrophilic dye. It is hypothesized that by increasing hydrophobicity, dyes may aggregate closer, thus improving coupling. This has been demonstrated in a set of squaraine dyes modified with hydrophobic substituents [70], and the values of log(P_o/w_) for the hydrophobic squaraine dyes are similar to those for the Cy5 derivatives. Comparing the Cy5 derivatives, Cy3, Cy5.5, and Cy7, Cy5-hex is the most hydrophobic and Cy3 is the least hydrophobic, closely followed by Cy5-CN. Most of the 15 dyes chosen from the ML Regressor model predictions exhibit hydrophobicity similar to that of Cy5. Three dyes are hydrophilic (dyes 2, 4, and 13), with dye 13 being the most hydrophilic. These three dyes also exhibit relatively low μ values compared to the rest of the dataset, indicating that they may not be suitable for excitonic applications that require close inter-dye separations and large transition dipole moment couplings. Conversely, dyes 7, 12, and 15 exhibit the most positive log(P_o/w_) values, meaning they are estimated to be the most hydrophobic. Furthermore, dyes 7, 12, and 15 have μ values within 25% of that for Cy5, making those dyes suitable for excitonic applications. Dyes 3 and 5 have log(P_o/w_) values slightly larger than that of Cy5, and μ values about 5 D larger than Cy5. Overall, based on our criteria—a large ε (indicating large μ) and a large positive log(P_o/w_)—dyes 3 and 5 are the most promising candidates in the dataset for excitonic applications.

### 3.2. Molecular Dynamics Simulations of Dye Aggregate–DNA Duplex Interactions

To study the effects of DNA on the dye orientations, 1 μs MD simulations were performed with two dyes covalently bound to the backbone of DNA duplexes via dual phosphoramidite linkers. In our study, we started with commercially available Cy5 and Cy5.5 as reference dyes to guide other dye candidate selection. Our research group experimentally demonstrated that Cy5 can exhibit aggregation, strong absorption, and excitonic coupling when attached to DNA duplexes and Holliday Junctions [27,28,29]. Due to its similar structure to Cy5, Cy5.5 should exhibit similar properties [38]. The extra aryl groups on Cy5.5 extend the conjugation and add to the size of the molecule, which could affect dye-packing and make $\mu_{\mathrm{Cy}5.5}$ slightly larger than $\mu_{\mathrm{Cy}5}$, as shown in Table 1. Furthermore, as shown in Figure 6, log(P_o/w_) for Cy5.5 is 40% larger than that for Cy5, indicating that Cy5.5 might pack closer than Cy5 when aggregated. Thus, we chose a Cy5 dimer and a Cy5.5 dimer for MD simulations as a benchmark for comparison with future simulations of other selected dyes. 

The simulations were performed in water at a 1 atm pressure and 300 K. Dye orientations were quantified using the orientation factor, κ, calculated using Equation (3). Dimer exciton exchange energies, |J|, were quantified using Equation (4) with inputs from TD-DFT for the transition dipole moments, where $\mu_{\mathrm{Cy}5}=15.35\;D$ and $\mu_{\mathrm{Cy}5.5}=15.57\;D$, as shown in Table 1. The vectors corresponding to μ were found to primarily reside on the long axis of the dyes, and thus, the values of $r_{i}$ and $s_{i}$ in Equation (4) were chosen as the centers of the terminal aryl groups of the dyes.

**Figure 7:**
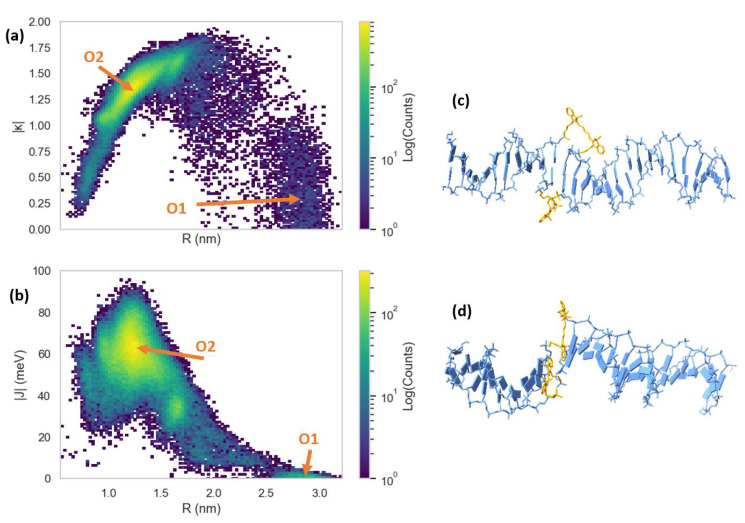
Heatmap plots of (**a**) orientation factor (|κ|) and (**b**) exciton exchange energy (|J|) versus dye center-to-center distances (R) for the 900 ns Cy5 dimer–DNA duplex molecular dynamics (MD) trajectory. Snapshots of the structural configurations of (**c**) pre-intercalation (corresponding to region O1) and (**d**) post-intercalation (corresponding to region O2) that represent regions in the |J| vs. R heatmap. The DNA duplex is shown in blue and the Cy5 dyes and linkers are shown in orange.

**Figure 8:**
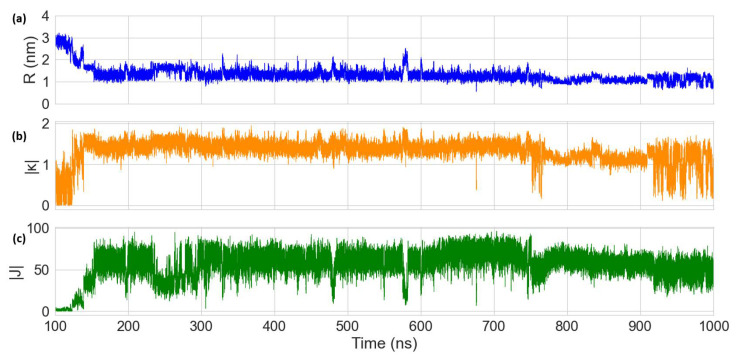
Plots of (**a**) dye center-to-center distance (R), (**b**) orientation factor (|κ|), and (**c**) exciton exchange energy (|J|) versus time for the 900 ns Cy5 dimer–DNA duplex MD trajectory. The first 100 ns of the simulation were treated as an equilibration and are therefore excluded.

The MD results for the Cy5 dimer attached to a DNA duplex are presented in Figure 7 and Figure 8. Figure 7a shows a heatmap plot of |κ| versus R, and Figure 7b shows a heatmap plot of |J| versus R for the 900 ns of data collection. Based on the |κ| values shown in Figure 7a, there are two distinct dimer orientations, labeled “O1” and “O2”. The approximate |J| regions corresponding to O1 and O2 are also shown in Figure 7b. As shown in Figure 7c, O1 corresponds to where the dyes are located outside of the duplex (i.e., non-intercalated). This orientation has a |κ| value ranging approximately from 0–1 (oblique and H-like aggregate) and an R of approximately 2.5–3.0 nm, which results in a relatively low |J| of less than 20 meV. Examining Figure 8, a shift in the orientation of the dyes occurs after around 150 ns of simulation, where the dyes re-orient and intercalate into the base-stack region of the DNA. This change in orientation results in a mostly head-to-tail (J-like) configuration with some obliqueness, corresponding to a |κ| of about 1.25–1.5 and an R of about 0.9–1.5 nm, as indicated by O2 and represented in Figure 7d. This more closely spaced orientation results in a larger |J| of roughly 40–80 meV. Since |J| was relatively stable after 200 ns, all dimer orientations beyond that time were averaged, yielding |κ|=1.35 ± 0.22 and R =1.26 ± 0.21 nm. Thus, averaging |J|  over this period of time results in |J|=58.28±12.74 meV. The post-200 ns average values of R and |J| agree well with the experimentally derived values of R =1.32 nm and |J|=48 meV, as obtained by Huff et al. [29]. Furthermore, they determined the dimer to have a similar orientation, with a red-shift in the main absorption peak observed for the dimer relative to the monomer, indicating a mostly J-like orientation [29]. The ~10 meV larger |J| value obtained from MD might be caused by a slight overestimation of $\mu_{\mathrm{Cy}5}$ using TD-DFT, which is shown to be ~2 D larger than the $\mu_{\mathrm{Cy}5}$ obtained from experimental measurements [29,46].

**Figure 9:**
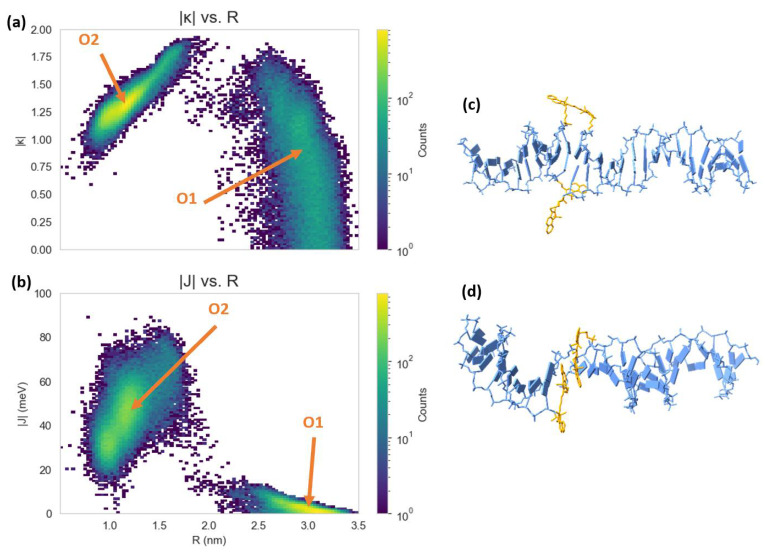
Heatmap plots of (**a**) orientation factor ((|κ|)), and (**b**) exciton exchange energy (|J|) versus dye center-to-center distances (R) for the 900 ns Cy5.5 dimer–DNA duplex MD trajectory. Snapshots of the structural configurations of (**c**) pre-intercalation (corresponding to region O1) and (**d**) post-intercalation (corresponding to region O2) that represent peaks in the |J| vs. R heatmap. The DNA duplex is shown in blue and the Cy5.5 dyes and linkers are shown in orange.

Compared to the Cy5 dimer, the Cy5.5 dimer exhibits a similar trajectory. The Cy5.5 dyes were initialized, outside of the DNA duplex (i.e., non-intercalated). After about 400 ns, the dyes intercalated, reducing R and increasing |κ| (more J-like) and |J|. The two regions corresponding to the non-intercalated and intercalated states of the Cy5.5 dimer are labeled as “O1” and “O2” in Figure 9a,b, which are represented in the snapshots in Figure 9c,d. The O1 region of the Cy5.5 dimer has an R that ranges from about 2.2–3.2 nm. However, |κ| for the Cy5.5 dimer has a range of roughly 0–1.5, which is larger than that for the Cy5 dimer. However, |J| for the O1 region of the Cy5.5 dimer is between 0–15 meV, comparable to that of the Cy5 dimer. The ranges of R and |κ| of the O2 region of Cy5.5 are roughly 0.8–1.7 nm and 1.2–1.75, respectively, similar to that of the Cy5 dimer. This range of orientations results in a |J| range of about 30–60 meV, slightly smaller than that of the Cy5 dimer.

**Figure 10:**
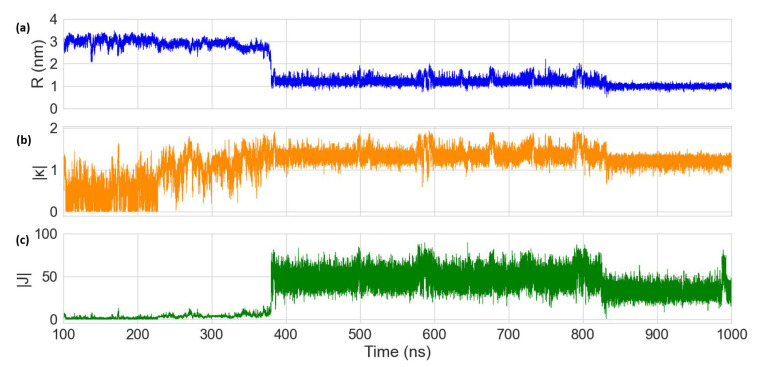
Plots of (**a**) dye center-to-center distance (R), (**b**) orientation factor (|κ|), and (**c**) exciton exchange energy (|J|) versus time for the 900 ns Cy5.5 dimer–DNA duplex MD trajectory. The first 100 ns of the simulation were treated as an equilibration and are therefore excluded.

Averaging the dimer orientations past 400 ns (after which |κ| and R are relatively stable, as shown in Figure 10) results in |κ|=1.33±0.15 and R =1.17±0.17 nm. Similarly, averaging |J| over this time period results in |J|=44.98 ± 11.6 meV. Even though the two dimers exhibit similar orientations, the smaller |J| for the Cy5.5 dimer might be caused by a small structure difference between Cy5.5 and Cy5. Cy5.5 is slightly larger and longer than Cy5 (by about 0.1 nm) due to the extra aryl groups at the two ends of the dye. Despite the smaller average R and similar orientations, the larger dye length of Cy5.5 compared to that of Cy5 results in a smaller pre-factor term $J_{0}$, leading to the smaller |J|. In a similar study, it was found that Cy5.5 homodimers attached to transverse strands on a DNA Holliday junction exhibited closer dye distances and larger |J| values than Cy5 homodimers [38]. A potential reason for this difference might be that the DNA Holliday junctions are more flexible than the duplexes, which allow dye orientations to promote larger J values.

## 4. Discussion

Based on the results shown in Figure 5, the developed Regressor is useful for the prediction of experimental ε trends. This was shown by predicting the ε of a dataset of over 18,000 molecules, from which 15 were identified using a combination of Regressor predictions and TD-DFT calculations. In general, according to desired criteria the developed ML model can quickly and accurately screen a large dataset of dyes for optimum ε for further study, which could save computation and experimental time.

There are improvements that can be made to the developed ML models. Primarily, more data would improve the chemical space on which the ML models would be trained, such as more chemical structures, as well as the inclusion of the solvents used. This is highlighted in Figure 1, which shows a disproportion in large versus small ε values in the training set. Furthermore, the present model could benefit from optimization and feature engineering, which could further reduce the computation time and help to improve the model by identifying more features that may better describe dye properties. Other types of ML models such as Support Vector Machine (SVM) algorithms could also be explored, which could potentially lead to better predictions. Finally, an improvement could be implemented by including human knowledge in the workflow so as to identify desirable structural features; this could be useful in interpreting data, improving efficiency, and enhancing model performance.

Examining Figure 5 and Table 1, the trends of the TD-DFT-calculated μ and the available experimentally determined ε mostly agree when considering the upper range of ε values. The deviations in ε compared to μ could be due to the solvent chosen for TD-DFT calculations (in our case, water) or the exchange–correlation functional used for the calculations. For example, our prior studies showed that the CAM-B3LYP functional produced slightly different μ values for similar dyes, however, the overall trend remained the same [46]. In the case of the Cy5 derivatives, the ε values might be affected by the specific DNA strand to which the dyes are attached, leading to the range in ε shown in Figure 5 for those dyes. This could also lead to some degree of disagreement with the TD-DFT calculations of μ (only considering free dyes in water) and the ML-predicted ε. Furthermore, in general, there is no strong correlation between the ML-predicted ε and TD-DFT μ for the 15 dyes shown in Table 1. This highlights the need for TD-DFT calculations as a validation for the initial dye screening using the ML Regressor, as the ML model is not 100% accurate. Studying the values of μ in Table 1, the main dye feature leading to a large μ is long conjugated chains, as is the case for dyes 3 and 5. The same trend is observed for Cy5 and Cy7, which have two and four more carbons in the conjugated chain compared to Cy3, respectively. Despite having similar structures, dye 10 has a ~1 D larger μ compared to dye 8, which might be caused by the Cl atom bonded to the polymethine chain in dye 8.

Examining Figure 6, the Cy5 derivatives modified with hydrophobic groups exhibit an enhanced hydrophobicity (as exemplified by more positive $\log\left(P_{o/w}\right)$ values), and the trend of $\log\left(P_{o/w}\right)$ follows the same trend obtained by Meares et al. who used the Percepta Platform to predict $\log\left(P_{o/w}\right)$ based on chemical structure [87]. Since these dyes are derived from the Cy5 dye, which has been shown to form aggregates when attached to DNA scaffolds [27,28,29], they are promising candidates for further studies with an aim of enhancing |J|. Examining the $\log\left(P_{o/w}\right)$ for the 15 ML-selected dyes, most exhibit values similar to Cy5 and its derivatives, with the notable exceptions of dyes 2, 4, 7, 12, 13, and 15. Dyes 2, 4, and 13 have a negative $\log\left(P_{o/w}\right)$ and are, therefore, expected to be hydrophilic. Dye 2 is structurally similar to dyes 1, 7, and 12, which have positive $\log\left(P_{o/w}\right)$ values. The hydrophilicity of dye 2 might be attributed to the Se atoms in the ring, which dyes 1, 7, and 12 do not contain. Dyes 4 and 13 contain S groups, which are known to increase hydrophilicity [101]. Dyes 7, 12, and 15 stand out as the most hydrophobic of the dyes tested and all belong to the same family of porphyrins, which are known to be hydrophobic [102,103]. Based on their relatively high μ and enhanced hydrophobicity, dyes 7, 12, and 15 (porphyrin-based dyes) are promising candidates for dye–DNA applications. Furthermore, it has been shown that porphyrin dyes are suitable for bonding to DNA and are able to intercalate between bases, which may be beneficial for dye aggregation in DNA [102].

Our MD simulations show that the Cy5 dimer and Cy5.5 dimer orient similarly when attached to the same positions on a DNA duplex. Comparing the simulation results for the Cy5 dimer to Huff et al., our predicted |J| and R are within 20% and 5%, respectively [29]. It should be noted that the results shown by Huff et al. were obtained using a program developed by our group based on the Kuhn–Renger–May (KRM) theory, which only considers absorbance and circular dichroism spectra to obtain dye orientations [29]. However, the KRM-based program does not provide information on the details of the DNA–dye orientations or the impact of the DNA on the dye orientation as our MD simulations do. Based on the MD results, one possible mechanism is dye intercalation into the DNA duplex from outside of the DNA backbone to form a J-dimer, which occurs for both Cy5 and Cy5.5 simulations. Our results highlight the effectiveness of MD simulations for predicting dye orientations and the excitonic properties of dyes bound to DNA duplexes. For future studies, we will continue applying MD to additional ML-selected dye aggregates in DNA scaffolds.

## 5. Conclusions

ML models were developed through training and validation on a set of 8802 molecules to predict ε based on dye structures. A Random Forest Classifier was developed with an accuracy of 97%, and based on the performance of the Classifier, a Random Forest Regressor was constructed. Comparing ML-predicted ε from the Regressor and experimental ε, the Regressor was found to have a maximum $R^{2}$ of 0.95. Using the Regressor, the ε values of molecules in a dataset of around 18,000 were predicted, and the top 100 dyes were used in TD-DFT calculations to calculate μ. Overall, 15 dyes were identified to have a relatively large μ comparable to Cy5. MD simulations were conducted on reference dyes (Cy5 and Cy5.5) to determine the dye dimer orientations and transition dipole moment couplings, |J|. For Cy5, the MD simulations were able to predict dye orientations and |J| within 20% of the experiment. The Cy5.5 dimer yielded similar results to the Cy5 dimer. The successful use of the combined ML and DFT/TD-DFT screening to identify dyes with a large ε and μ highlights the effectiveness of our workflow to screen numerous dyes for desired properties. The agreement of our MD simulations with the experiment show that we can accurately detect dye dimer properties when attached to DNA scaffolds, which is crucial to guide dye design and synthesis for excitonic applications.

## Figures and Tables

**Table 1:** ML-Regressor predicted extinction coefficient (ε) and TD-DFT-calculated transition dipole moments (μ) of dyes shown in Figure 4. Cartesian μ vector components along x, y, and z axis are provided in Table S1.

Dye	ML-Predicted ε $\times \;1000\;M^{-1}cm^{-1}$	TD-DFT μ, Debye
Cy3	126	12.25
Cy5	222	15.35
Cy5-CN	138	15.66
Cy5-NMe_2_	216	15.83
Cy5-Cl	214	15.74
Cy5-hex	110	16.22
Cy5-Peg	107	16.19
Cy5-tBu	224	15.90
Cy5.5	205	15.57
Cy7	235	17.62
1	307	9.08
2	288	7.84
3	265	20.25
4	240	7.99
5	235	20.17
6	229	14.00
7	227	10.66
8	227	15.49
9	226	14.68
10	223	16.19
11	222	14.00
12	218	11.26
13	216	9.52
14	212	9.34
15	210	10.48

## Data Availability

Select ML-predicted extinction coefficients, solvation energy, and transition dipole moment data presented in this study are available in the main text and in the Supplementary Materials. The data used to train and validate the machine learning models and the codes for the models will be published online soon.

## Supplementary Materials

The following supporting information can be downloaded at: https://www.mdpi.com/article/10.3390/molecules27113456/s1, Table S1: Transition dipole moment (μ) vector components of the dyes in Figure 4; Table S2: Gibbs free energies of solvation (${\Delta G}_{\mathrm{solv}}$) of the dyes in Figure 4 in water and n-octanol implicit solvents.

## Sample Availability

Samples of the compounds are not available from the authors.

## References

[1] Ilina K., MacCuaig W.M., Laramie M., Jeouty J.N., McNally L.R., Henary M. (2020). Squaraine Dyes: Molecular Design for Different Applications and Remaining Challenges. Bioconjug. Chem..

[2] Umezawa K., Citterio D., Suzuki K. (2014). New Trends in Near-Infrared Fluorophores for Bioimaging. Anal. Sci..

[3] Scholes G.D., Fleming G.R., Olaya-Castro A., Van Grondelle R. (2011). Lessons from nature about solar light harvesting. Nat. Chem..

[4] Collado-Fregoso E., Boufflet P., Fei Z., Gann E., Ashraf S., Li Z., Mcneill C.R., Durrant J.R., Heeney M. (2015). Increased Exciton Dipole Moment Translates into Charge-Transfer Excitons in Thiophene-Fluorinated Low-Bandgap Polymers for Organic Photovoltaic Applications. Chem. Mater..

[5] Markov R.V., Plekhanov A.I., Shelkovnikov V.V., Knoester J. (2000). Giant Nonlinear Optical Response of Interacting One-Dimensional Frenkel Excitons in Molecular Aggregates. Phys. Status Solidi.

[6] Kellis D.L., Sarter C., Cannon B.L., Davis P.H., Graugnard E., Lee J., Pensack R.D., Kolmar T., Jäschke A., Yurke B. (2019). An All-Optical Excitonic Switch Operated in the Liquid and Solid Phases. ACS Nano.

[7] Cannon B.L., Kellis D.L., Davis P.H., Lee J., Kuang W., Hughes W.L., Graugnard E., Yurke B., Knowlton W.B. (2015). Excitonic AND Logic Gates on DNA Brick Nanobreadboards. ACS Photonics.

[8] Outeiral C., Strahm M., Shi J., Morris G.M., Benjamin S.C., Deane C.M. (2021). The prospects of quantum computing in computational molecular biology. Wiley Interdiscip. Rev. Comput. Mol. Sci..

[9] Wasielewski M.R., Forbes M.D.E., Frank N.L., Kowalski K., Scholes G.D., Yuen-Zhou J., Baldo M.A., Freedman D.E., Goldsmith R.H., Goodson T. (2020). Exploiting chemistry and molecular systems for quantum information science. Nat. Rev. Chem..

[10] Lewis J.E., Maroncelli M. (1998). On the (uninteresting) dependence of the absorption and emission transition moments of coumarin 153 on solvent. Chem. Phys. Lett..

[11] Chako N.Q. (1934). Absorption of light in organic compounds. J. Chem. Phys..

[12] Marciniak H., Auerhammer N., Ricker S., Schmiedel A., Holzapfel M., Lambert C. (2019). Reduction of the Fluorescence Transition Dipole Moment by Excitation Localization in a Vibronically Coupled Squaraine Dimer. J. Phys. Chem. C.

[13] Namuangruk S., Fukuda R., Ehara M., Meeprasert J., Khanasa T., Morada S., Kaewin T., Jungsuttiwong S., Sudyoadsuk T., Promarak V. (2012). D−D−π−A-Type Organic Dyes for Dye-Sensitized Solar Cells with a Potential for Direct Electron Injection and a High Extinction Coefficient: Synthesis, Characterization, and Theoretical Investigation. J. Phys. Chem. C.

[14] Zhang W., Wu Y., Zhu H., Chai Q., Liu J., Li H., Song X., Zhu W.-H. (2015). Rational Molecular Engineering of Indoline-Based D-A-π-A Organic Sensitizers for Long-Wavelength-Responsive Dye-Sensitized Solar Cells. ACS Appl. Mater. Interfaces.

[15] Song X., Yang X., Wang H., An J., Yu Z., Wang X., Hagfeldt A., Sun L. (2019). Improving energy transfer efficiency of dye-sensitized solar cell by fine tuning of dye planarity. Sol. Energy.

[16] Sik Yoon W., Won Kim D., Park J.-M., Cho I., Kyu Kwon O., Ryeol Whang D., Hong Kim J., Park J.-H., Young Park S. (2016). A Novel Bis-Lactam Acceptor with Outstanding Molar Extinction Coefficient and Structural Planarity for Donor−Acceptor Type Conjugated Polymer. Macromolecules.

[17] Che Y., Perepichka D.F. (2021). Quantifying Planarity in the Design of Organic Electronic Materials. Angew. Chem.-Int. Ed..

[18] Engel G.S., Calhoun T.R., Read E.L., Ahn T.K., Mančal T., Cheng Y.C., Blankenship R.E., Fleming G.R. (2007). Evidence for wavelike energy transfer through quantum coherence in photosynthetic systems. Nature.

[19] Mirkovic T., Ostroumov E.E., Anna J.M., Van Grondelle R., Govindjee, Scholes G.D. (2017). Light absorption and energy transfer in the antenna complexes of photosynthetic organisms. Chem. Rev..

[20] Lim J.M., Kim P., Yoon M.C., Sung J., Dehm V., Chen Z., Würthner F., Kim D. (2013). Exciton delocalization and dynamics in helical π-stacks of self-assembled perylene bisimides. Chem. Sci..

[21] Bialas D., Zitzler-Kunkel A., Kirchner E., Schmidt D., Würthner F. (2016). Structural and quantum chemical analysis of exciton coupling in homo-and heteroaggregate stacks of merocyanines. Nat. Commun..

[22] Kasha M. (1963). Energy Transfer Mechanisms and the Molecular Exciton Model for Molecular Aggregates. Radiat. Res..

[23] Abramavicius D., Palmieri B., Mukamel S. (2009). Extracting single and two-exciton couplings in photosynthetic complexes by coherent two-dimensional electronic spectra. Chem. Phys..

[24] Kasha M., Rawls H.R., Ashraf El-Bayoumi M. (1965). The Exciton Model in Molecular Spectroscopy. Pure Appl. Chem..

[25] Davydov A.S. (1948). Theory of Absorption Spectra of Molecular Crystals. Transl. Repr. Zh. Eksp. Teor. Fiz..

[26] Davydov A.S. (1964). The Theory of Molecular Excitons. Sov. Phys. Uspekhi.

[27] Cannon B.L., Kellis D.L., Patten L.K., Davis P.H., Lee J., Graugnard E., Yurke B., Knowlton W.B. (2017). Coherent Exciton Delocalization in a Two-State DNA-Templated Dye Aggregate System. J. Phys. Chem. A.

[28] Cannon B.L., Patten L.K., Kellis D.L., Davis P.H., Lee J., Graugnard E., Yurke B., Knowlton W.B. (2018). Large Davydov Splitting and Strong Fluorescence Suppression: An Investigation of Exciton Delocalization in DNA-Templated Holliday Junction Dye Aggregates. J. Phys. Chem. A.

[29] Huff J.S., Turner D.B., Mass O.A., Patten L.K., Wilson C.K., Roy S.K., Barclay M.S., Yurke B., Knowlton W.B., Davis P.H. (2021). Excited-State Lifetimes of DNA-Templated Cyanine Dimer, Trimer, and Tetramer Aggregates: The Role of Exciton Delocalization, Dye Separation, and DNA Heterogeneity. J. Phys. Chem. B.

[30] Hart S.M., Chen W.J., Banal J.L., Bricker W.P., Dodin A., Markova L., Vyborna Y., Willard A.P., Häner R., Bathe M. (2021). Engineering couplings for exciton transport using synthetic DNA scaffolds. Chem.

[31] Mass O.A., Wilson C.K., Roy S.K., Barclay M.S., Patten L.K., Terpetschnig E.A., Lee J., Pensack R.D., Yurke B., Knowlton W.B. (2020). Exciton Delocalization in Indolenine Squaraine Aggregates Templated by DNA Holliday Junction Scaffolds. J. Phys. Chem. B.

[32] Barclay M.S., Roy S.K., Huff J.S., Mass O.A., Turner D.B., Wilson C.K., Kellis D.L., Terpetschnig E.A., Lee J., Davis P.H. (2021). Rotaxane rings promote oblique packing and extended lifetimes in DNA-templated molecular dye aggregates. Commun. Chem..

[33] Banal J.L., Kondo T., Veneziano R., Bathe M., Schlau-Cohen G.S. (2017). Photophysics of J-Aggregate-Mediated Energy Transfer on DNA. J. Phys. Chem. Lett..

[34] Markova L.I., Malinovskii V.L., Patsenker L.D., Häner R. (2013). J- vs. H-type assembly: Pentamethine cyanine (Cy5) as a near-IR chiroptical reporter. Chem. Commun..

[35] Kringle L., Sawaya N.P.D., Widom J., Adams C., Raymer M.G., Aspuru-Guzik A., Marcus A.H. (2018). Temperature-dependent conformations of exciton-coupled Cy3 dimers in double-stranded DNA. J. Chem. Phys..

[36] Seifert J.L., Connor R.E., Kushon S.A., Wang M., Armitage B.A. (1999). Spontaneous Assembly of Helical Cyanine Dye Aggregates on DNA Nanotemplates. J. Am. Chem. Soc..

[37] Garoff R.A., Litzinger E.A., Connor R.E., Fishman I., Armitage B.A. (2002). Helical Aggregation of Cyanine Dyes on DNA Templates: Effect of Dye Structure on Formation of Homo-and Heteroaggregates. Langmuir.

[38] Chowdhury A.U., Díaz S.A., Huff J.S., Barclay M.S., Chiriboga M., Ellis G.A., Mathur D., Patten L.K., Sup A., Hallstrom N. (2022). Tuning between Quenching and Energy Transfer in DNA-Templated Heterodimer Aggregates. J. Phys. Chem. Lett..

[39] Roy S.K., Mass O.A., Kellis D.L., Wilson C.K., Hall J.A., Yurke B., Knowlton W.B. (2021). Exciton Delocalization and Scaffold Stability in Bridged Nucleotide-Substituted, DNA Duplex-Templated Cyanine Aggregates. J. Phys. Chem. B.

[40] Jelley E.E. (1936). Spectral absorption and fluorescence of dyes in the molecular state. Nature.

[41] Abou-Hatab S., Spata V.A., Matsika S. (2017). Substituent Effects on the Absorption and Fluorescence Properties of Anthracene. J. Phys. Chem. A.

[42] Cervantes-Navarro F., Glossman-Mitnik D. (2012). DFT study of the effect of substituents on the absorption and emission spectra of Indigo. Chem. Cent. J..

[43] Tai C.K., Chen Y.J., Chang H.W., Yeh P.L., Wang B.C. (2011). DFT and TD-DFT investigations of metal-free dye sensitizers for solar cells: Effects of electron donors and π-conjugated linker. Comput. Theor. Chem..

[44] Inostroza N., Mendizabal F., Arratia-Pérez R., Orellana C., Linares-Flores C. (2016). Improvement of photovoltaic performance by substituent effect of donor and acceptor structure of TPA-based dye-sensitized solar cells. J. Mol. Model..

[45] Barcenas G., Biaggne A., Mass O.A., Wilson C.K., Obukhova O.M., Kolosova O.S., Tatarets A.L., Terpetschnig E., Pensack R.D., Lee J. (2021). First-principles studies of substituent effects on squaraine dyes. RSC Adv..

[46] Biaggne A., Knowlton W.B., Yurke B., Lee J., Li L. (2021). Substituent Effects on the Solubility and Electronic Properties of the Cyanine Dye Cy5: Density Functional and Time-Dependent Density Functional Theory Calculations. Molecules.

[47] Mathur D., Kim Y.C., Díaz S.A., Cunningham P.D., Rolczynski B.S., Ancona M.G., Medintz I.L., Melinger J.S. (2021). Can a DNA Origami Structure Constrain the Position and Orientation of an Attached Dye Molecule?. J. Phys. Chem. C.

[48] Cunningham P.D., Kim Y.C., Díaz S.A., Buckhout-White S., Mathur D., Medintz I.L., Melinger J.S. (2018). Optical Properties of Vibronically Coupled Cy3 Dimers on DNA Scaffolds. J. Phys. Chem. B.

[49] Stennett E.M.S., Ma N., van der Vaart A., Levitus M. (2014). Photophysical and Dynamical Properties of Doubly Linked Cy3–DNA Constructs. J. Phys. Chem. B.

[50] Nicoli F., Roos M.K., Hemmig E.A., Di Antonio M., de Vivie-Riedle R., Liedl T. (2016). Proximity-Induced H-Aggregation of Cyanine Dyes on DNA-Duplexes. J. Phys. Chem. A.

[51] Kang B., Seok C., Lee J. (2020). Prediction of Molecular Electronic Transitions Using Random Forests. J. Chem. Inf. Model..

[52] Joung J.F., Han M., Hwang J., Jeong M., Choi D.H., Park S. (2021). Deep Learning Optical Spectroscopy Based on Experimental Database: Potential Applications to Molecular Design. JACS Au.

[53] Beard E.J., Sivaraman G., Vázquez-Mayagoitia Á., Vishwanath V., Cole J.M. (2019). Comparative dataset of experimental and computational attributes of UV/vis absorption spectra. Sci. Data.

[54] Taniguchi M., Du H., Lindsey J.S. (2018). PhotochemCAD 3: Diverse Modules for Photophysical Calculations with Multiple Spectral Databases. Photochem. Photobiol..

[55] Nagasawa S., Al-Naamani E., Saeki A. (2018). Computer-Aided Screening of Conjugated Polymers for Organic Solar Cell: Classification by Random Forest. J. Phys. Chem. Lett..

[56] Cai J., Chu X., Xu K., Li H., Wei J. (2020). Machine learning-driven new material discovery. Nanoscale Adv..

[57] Joung J.F., Han M., Jeong M., Park S. (2020). Experimental database of optical properties of organic compounds. Sci. Data.

[58] Dyomics GmbH. https://dyomics.com/en/.

[59] RDKit: Open-Source Cheminformatics. https://www.rdkit.org/.

[60] Zhao Y., Truhlar D.G. (2008). The M06 suite of density functionals for main group thermochemistry, thermochemical kinetics, noncovalent interactions, excited states, and transition elements: Two new functionals and systematic testing of four M06-class functionals and 12 other function. Theor. Chem. Acc..

[61] Kawauchi S., Antonov L., Okuno Y. (2014). Prediction of the color of dyes by using time-dependent density functional theory (TD-DFT). Bulg. Chem. Commun..

[62] Charaf-Eddin A., Planchat A., Mennucci B., Adamo C., Jacquemin D. (2013). Choosing a functional for computing absorption and fluorescence band shapes with TD-DFT. J. Chem. Theory Comput..

[63] Jacquemin D., Zhao Y., Valero R., Adamo C., Ciofini I., Truhlar D.G. (2012). Verdict: Time-dependent density functional theory “not guilty” of large errors for cyanines. J. Chem. Theory Comput..

[64] Cancès E., Mennucci B., Tomasi J. (1997). A new integral equation formalism for the polarizable continuum model: Theoretical background and applications to Isotropic and anisotropic dielectrics. J. Chem. Phys..

[65] Tomasi J., Mennucci B., Cammi R. (2005). Quantum mechanical continuum solvation models. Chem. Rev..

[66] Selvam K., Gandhi S., Krishnamurty S., Gopalakrishnan G. (2019). Effect of substitution on the excited state photophysical and spectral properties of boron difluoride curcumin complex dye and their derivatives: A time dependent-DFT study. J. Photochem. Photobiol. B Biol..

[67] Heid E., Hunt P.A., Schröder C. (2018). Evaluating excited state atomic polarizabilities of chromophores. Phys. Chem. Chem. Phys..

[68] Fothergill J.W., Hernandez A.C., Knowlton W.B., Yurke B., Li L. (2018). Ab Initio Studies of Exciton Interactions of Cy5 Dyes. J. Phys. Chem. A.

[69] Garrido N.M., Economou I.G., Queimada A.J., Jorge M., Macedo E.A. (2012). Prediction of the n-Hexane/Water and 1-Octanol/Water Partition Coefficients for Environmentally Relevant Compounds using Molecular Simulation. AIChE J..

[70] Mass O.A., Wilson C.K., Barcenas G., Terpetschnig E.A., Obukhova O.M., Kolosova O.S., Tatarets A.L., Li L., Yurke B., Knowlton W.B. (2022). Influence of Hydrophobicity on Excitonic Coupling in DNA-Templated Indolenine Squaraine Dye Aggregates. J. Phys. Chem. C.

[71] Mananghaya M.R., Santos G.N., Yu D.N. (2017). Solubility of amide functionalized single wall carbon nanotubes: A quantum mechanical study. J. Mol. Liq..

[72] Marenich A.V., Cramer C.J., Truhlar D.G. (2009). Universal solvation model based on solute electron density and on a continuum model of the solvent defined by the bulk dielectric constant and atomic surface tensions. J. Phys. Chem. B.

[73] Zhang J., Zhang H., Wu T., Wang Q., Van Der Spoel D. (2017). Comparison of Implicit and Explicit Solvent Models for the Calculation of Solvation Free Energy in Organic Solvents. J. Chem. Theory Comput..

[74] Frisch M.J., Trucks G.W., Schlegel H.B., Scuseria G.E., Robb M.A., Cheeseman J.R., Scalmani G., Barone V., Petersson G.A., Nakatsuji H. (2016). Gaussian 16.

[75] Van Der Spoel D., Lindahl E., Hess B., Groenhof G., Mark A.E., Berendsen H.J.C. (2005). GROMACS: Fast, flexible, and free. J. Comput. Chem..

[76] Pettersen E.F., Goddard T.D., Huang C.C., Meng E.C., Couch G.S., Croll T.I., Morris J.H., Ferrin T.E. (2021). UCSF ChimeraX: Structure visualization for researchers, educators, and developers. Protein Sci..

[77] Galindo-Murillo R., Robertson J.C., Zgarbová M., Šponer J., Otyepka M., Jurečka P., Cheatham T.E. (2016). Assessing the Current State of Amber Force Field Modifications for DNA. J. Chem. Theory Comput..

[78] Yoo J., Aksimentiev A. (2012). Improved parametrization of Li^+^, Na^+^, K^+^, and Mg^2+^ ions for all-atom molecular dynamics simulations of nucleic acid systems. J. Phys. Chem. Lett..

[79] Wang J., Wolf R.M., Caldwell J.W., Kollman P.A., Case D.A. (2004). Development and testing of a general Amber force field. J. Comput. Chem..

[80] Bayly C.I., Cieplak P., Cornell W.D., Kollman P.A. (1993). A well-behaved electrostatic potential based method using charge restraints for deriving atomic charges: The RESP model. J. Phys. Chem..

[81] Jorgensen W.L., Chandrasekhar J., Madura J.D., Impey R.W., Klein M.L. (1983). Comparison of simple potential functions for simulating liquid water. J. Chem. Phys..

[82] Hess B., Bekker H., Berendsen H.J.C., Fraaije J.G.E.M. (1997). LINCS: A linear constraint solver for molecular simulations. J. Comput. Chem..

[83] Bussi G., Donadio D., Parrinello M. (2007). Canonical sampling through velocity rescaling. J. Chem. Phys..

[84] Parrinello M., Rahman A. (1981). Polymorphic transitions in single crystals: A new molecular dynamics method. J. Appl. Phys..

[85] Czikklely V., Forsterling H.D., Kuhn H. (1970). Extended dipole model for aggregates of dye molecules. Chem. Phys. Lett..

[86] Mujumdar R.B., Ernst L.A., Mujumdar S.R., Lewis C.J., Waggoner A.S. (1993). Cyanine Dye Labeling Reagents: Sulfoindocyanine Succinimidyl Esters. Bioconjug. Chem..

[87] Meares A., Susumu K., Mathur D., Lee S.H., Mass O.A., Lee J., Pensack R.D., Yurke B., Knowlton W.B., Melinger J.S. (2022). Synthesis of Substituted Cy5 Phosphoramidite Derivatives and Their Incorporation into Oligonucleotides Using Automated DNA Synthesis. ACS Omega.

[88] Kim S., Chen J., Cheng T., Gindulyte A., He J., He S., Li Q., Shoemaker B.A., Thiessen P.A., Yu B. (2021). PubChem in 2021: New data content and improved web interfaces. Nucleic Acids Res..

[89] Levitus M., Ranjit S. (2011). Cyanine dyes in biophysical research: The photophysics of polymethine fluorescent dyes in biomolecular environments. Q. Rev. Biophys..

[90] Zeyada H.M., Makhlouf M.M., Behairy A.S., Nasher M.A. (2016). Fabrication, electrical transport mechanisms and photovoltaic properties of methyl violet 2B/n-Si hybrid organic/inorganic solar cell. Microelectron. Eng..

[91] Chen J., Gao Y., Xu Y., Xu F., Zhang Q., Lu X. (2019). Theoretical study of novel porphyrin D-π-A conjugated organic dye sensitizer in solar cells. Mater. Chem. Phys..

[92] Li L.L., Diau E.W.G. (2013). Porphyrin-sensitized solar cells. Chem. Soc. Rev..

[93] Sameiro M., Gonçalves T. (2009). Fluorescent labeling of biomolecules with organic probes. Chem. Rev..

[94] Pan X., Huang S., Zhu B., Xia R., Peng X. (2020). All-porphyrin organic solar cells. Dye Pigment..

[95] Wan Y., Stradomska A., Knoester J., Huang L. (2017). Direct Imaging of Exciton Transport in Tubular Porphyrin Aggregates by Ultrafast Microscopy. J. Am. Chem. Soc..

[96] Bricks J.L., Slominskii Y.L., Panas I.D., Demchenko A.P. (2018). Fluorescent J-aggregates of cyanine dyes: Basic research and applications review. Methods Appl. Fluoresc..

[97] AAT Bioquest. https://www.aatbio.com/.

[98] Lumiprobe. https://www.lumiprobe.com/.

[99] Glen Research. https://www.glenresearch.com/.

[100] Interchim. https://www.interchim.com/.

[101] Markova L.I., Terpetschnig E.A., Patsenker L.D. (2013). Comparison of a series of hydrophilic squaraine and cyanine dyes for use as biological labels. Dye Pigment..

[102] Murashima T., Hayata K., Saiki Y., Matsui J., Miyoshi D., Yamada T., Miyazawa T., Sugimoto N. (2007). Synthesis, structure and thermal stability of fully hydrophobic porphyrin-DNA conjugates. Tetrahedron Lett..

[103] Ben-Dror S., Bronshtein I., Wiehe A., Röder B., Senge M.O., Ehrenberg B. (2006). On the Correlation Between Hydrophobicity, Liposome Binding and Cellular Uptake of Porphyrin Sensitizers. Photochem. Photobiol..

