# Supplementary material for: Data-Driven and Multiscale Modeling of DNA-Templated Dye Aggregates
Source: Molecules. 2022 May 27;27(11):3456. doi: 10.3390/molecules27113456 (PMC9182218; doi:10.3390/molecules27113456)
Supplement: Supplementary file 1 [file molecules-27-03456-s001.zip › molecules-1732252-supplementary.pdf]

Table S1. Cartesian transition dipole moment (μ) vector components of dyes in implicit water solvent. Vertical excited state calculations were done using the ground state geometry. Dye structures are shown in Figure 4 of the main text.

**Dye**	**μ_x (Debye)**	**μ_y (Debye)**	**μ_z (Debye)**
**Cy3**	12.25	0.00	0.00
**Cy5**	15.35	0.00	0.00
**Cy5-CN**	15.66	-0.24	0.00
**Cy5-NMe2**	15.81	-0.74	-0.01
**Cy5-Cl**	-15.74	0.00	0.00
**Cy5-hex**	16.22	0.00	0.00
**Cy5-Peg**	-16.19	0.00	0.00
**Cy5-tBu**	-15.90	0.00	0.00
**Cy5.5**	-15.57	0.00	0.00
**Cy7**	17.62	0.00	0.00
**1**	1.03	8.98	0.86
**2**	-7.81	0.43	-0.50
**3**	-20.25	0.00	0.10
**4**	7.97	0.42	-0.25
**5**	-20.17	0.00	0.32
**6**	13.99	0.21	0.13
**7**	4.68	9.57	0.49
**8**	-15.45	1.08	0.20
**9**	-14.67	-0.46	0.13
**10**	16.15	1.02	0.23
**11**	-14.00	0.30	-0.03
**12**	-10.84	-2.63	1.56
**13**	-3.47	7.98	-3.88
**14**	9.23	-1.43	0.00
**15**	8.51	-6.10	-0.27

Table S2. Gibbs free energy of solvation (ΔG_{solv}) of dyes in implicit water and n-octanol solvents. The values of ΔG_{solv} were determined using Equation (2) with the dye geometry relaxed in implicit solvent and vacuum. Dye structures are shown in Figure 4 of the main text.

**Dye**	$\Delta G_{solv}^{n-octanol}$ **(Hartree)**	ΔG_{solv}^{water} **(Hartree)**
**Cy3**	-0.0743	-0.0655
**Cy5**	-0.0735	-0.0626
**Cy5-CN**	-0.0831	-0.0739
**Cy5-NMe2**	-0.0728	-0.0618
**Cy5-Cl**	-0.0812	-0.0690
**Cy5-hex**	-0.0806	-0.0597
**Cy5-Peg**	-0.0966	-0.0845
**Cy5-tBu**	-0.0734	-0.0567
**Cy5.5**	-0.0785	-0.0650
**Cy7**	-0.0740	-0.0596
**1**	-0.0329	-0.0169
**2**	-0.0845	-0.0886
**3**	-0.0738	-0.0612
**4**	-0.0251	-0.0263
**5**	-0.0757	-0.0632
**6**	-0.0737	-0.0625
**7**	-0.0344	-0.0131
**8**	-0.0780	-0.0685
**9**	-0.0479	-0.0377
**10**	-0.0745	-0.0653
**11**	-0.0715	-0.0587
**12**	-0.0478	-0.0201
**13**	-0.0431	-0.0561
**14**	-0.0260	-0.0225
**15**	-0.0433	-0.0189
